# E3 Ubiquitin Ligase *Uhrf2* Knockout Reveals a Critical Role in Social Behavior and Synaptic Plasticity in the Hippocampus

**DOI:** 10.3390/ijms25031543

**Published:** 2024-01-26

**Authors:** Yinghan Zhuang, Chuhan Li, Fang Zhao, Yan Yan, Hongjie Pan, Jianmin Zhan, Thomas Behnisch

**Affiliations:** 1 State Key Laboratory of Medical Neurobiology and MOE Frontiers Center for Brain Science, Institutes of Brain Science, Fudan University, Shanghai 200032, China; 2 National Health Commission (NHC) Key Laboratory of Reproduction Regulation, Shanghai Institute of Planned Parenthood Research, Fudan University, Shanghai 200032, China

**Keywords:** E3 ubiquitin ligase, UHRF2, social memory, CA2, synaptic plasticity, hippocampus

## Abstract

The hippocampal formation, particularly the CA2 subregion, is critical for social memory formation and memory processing, relying on synaptic plasticity—a fundamental mechanism by which synapses strengthen. Given the role of the ubiquitin–proteasome system (UPS) in various nervous system processes, including learning and memory, we were particularly interested in exploring the involvement of RING-type ubiquitin E3 ligases, such as UHRF2 (NIRF), in social behavior and synaptic plasticity. Our results revealed altered social behavior in mice with systemic *Uhrf2* knockout, including changes in nest building, tube dominance, and the three-chamber social novelty test. In *Uhrf2* knockout mice, the entorhinal cortex-CA2 circuit showed significant reductions in synaptic plasticity during paired-pulse facilitation and long-term potentiation, while the inability to evoke synaptic plasticity in the Schaffer-collateral CA2 synapses remained unaffected. These changes in synaptic plasticity correlated with significant changes in gene expression including genes related to vesicle trafficking and transcriptional regulation. The effects of *Uhrf2* knockout on synaptic plasticity and the observed gene expression changes highlight UHRF2 as a regulator of learning and memory processes at both the cellular and systemic levels. Targeting E3 ubiquitin ligases, such as UHRF2, may hold therapeutic potential for memory-related disorders, warranting further investigation.

## 1. Introduction

The hippocampus, a crucial brain region for memory formation, consists of distinct subregions with unique physiological properties that regulate different types of memory [1]. Among these subregions, Cornu Ammonis 1 (CA1) occupies a significant portion of the hippocampus and receives inputs from CA3 pyramidal neurons, as well as direct input from CA2, the medial entorhinal cortex (MEC), and the lateral entorhinal cortex (LEC) [2,3,4,5]. Cornu Ammonis 2 (CA2), located between CA1 and CA3, exhibits distinct molecular characteristics, including increased expression of specific genes and proteins such as RGS14, TCP4, ACTN2, and PTPN5 [6,7].

Recent studies have emphasized the critical role of the CA2 subregion in social memory formation and socio-cognitive information processing [8,9,10,11,12,13]. Notably, impairments in social memory have been observed when hippocampal CA2 neurons are inactivated, leading to the inability of animals to recognize familiar individuals [10,13]. Interestingly, studies have demonstrated that the inhibition of CA2 pyramidal neurons disrupts social memory, whereas their activation promotes both social memory and social aggression [10,13,14]. Moreover, the distinctive electrophysiological properties of CA2 excitatory neurons, characterized by their elevated negative resting membrane potentials, underscored the involvement of this region in social behavior [15]. Importantly, in addition to inputs from CA3, CA2 neurons receive afferent projections from layer II/III of the entorhinal cortex (EC) that synapse in the stratum lacunosum–moleculare. In this distal dendritic region, EC-CA2 synapses exhibit activity-dependent long-term potentiation (LTP) [16,17]. In contrast, proximal Schaffer collateral inputs (SC) from CA3 to CA2 do not support activity-dependent LTP [15,18], highlighting regional differences in the modulation of synaptic transmission by neural activity patterns [12].

While the morphological and physiological specializations underlying the role of CA2 role are becoming clearer, the specific molecular factors regulating social memory formation in this region remain less understood. One potential modulatory mechanism is protein turnover, which plays a pivotal role in neural development and plasticity [19,20,21]. The ubiquitin–proteasome system (UPS) is responsible for regulated protein degradation and is essential for synaptic plasticity and memory formation [22,23,24,25,26]. Dysregulation of the UPS contributes to synaptic dysfunction and memory impairments in Alzheimer’s disease (AD) and Parkinson’s disease (PD). Interestingly, targeted modulation of the UPS has shown promise in restoring impaired synaptic plasticity and memory in animal models of AD [27,28]. For example, overexpression of the ubiquitin E3 ligase Parkin, despite its association with PD pathogenesis, can counteract AD-related deficits in mice by ubiquitinating substrates [29], highlighting the potential of strategic UPS regulation as a therapeutic approach.

Given the central influence of protein regulation on cognition, elucidating the role of specific UPS components, such as the E3 ubiquitin ligase UHRF2, in hippocampus-dependent learning warrants exploration. UHRF2, encoded by the *Uhrf2* gene [30], induces protein degradation through its RING finger domain and regulates gene transcription through histone modification [31,32]. Initial findings suggest that *Uhrf2* deficiency may affect object recognition [33]. Intriguingly, while UPS components have been implicated in cognitive processes, UHRF2 remains uncharacterized in the context of social memory and CA2 synaptic plasticity. Thus, elucidating the influence of UHRF2-mediated ubiquitination on hippocampal circuitry may provide the first insights into how protein turnover mechanisms regulate this domain of memory formation.

To address these open questions, we conducted a study utilizing *Uhrf2* knockout mice to evaluate the role of UHRF2 in modulating social behavior and synaptic plasticity specifically in the CA2 hippocampal subregion. By combining behavioral assays, electrophysiology, and unbiased RNA sequencing, we uncovered novel regulatory functions of UHRF2 in social behavior, entorhinal cortex–CA2 synaptic potentiation, and the modulation of a variety of gene expression networks. Thus, by evaluating the functions of UHRF2 in hippocampal social behavior and plasticity, our findings advance our understanding of how ubiquitin-dependent protein regulation shapes the properties of the CA2 memory circuit.

## 2. Results

### 2.1. Altered Social Interactions of Uhrf2 Knockout Mice

Social behavior constitutes a fundamental trait in numerous species, facilitating adaptation to environmental pressures, promoting community stability, and appropriate responses to external cues [34]. Perturbations in social behavior manifest prominently across neurological disorders, including depression, autism, bipolar disorder, and dementia, thereby underscoring its overarching importance in cognition [35,36]. In their natural habitats, mice exhibit a diverse array of social behaviors, such as nest construction, evaluating potential mates, mutual grooming, and hierarchy development, which rely extensively on proficient social cognition and communication abilities. More specifically, the skill to build nests constitutes an essential capability in mice, providing thermal regulation, shelter, and a venue for reproduction [37]. Therefore, it is important to consider nest-building skills as a potential marker for cognitive function in mice. Lack of nesting behavior has been associated with cognitive deficits after brain injury, pharmacological intervention, or in transgenic mouse strains [38]. Taken together, the assessment of nest-building capacity provides vital insights into general social competence and cognition in mice.

**Figure 1:**
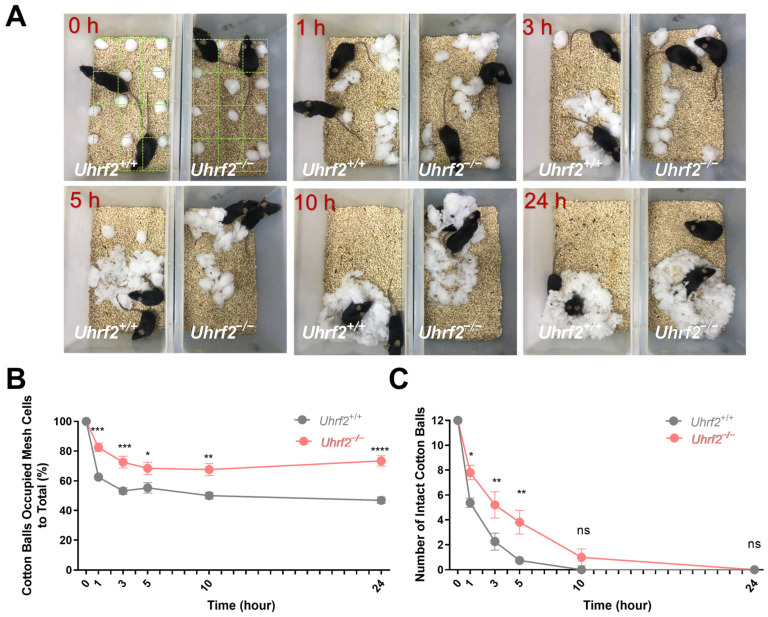
Quantification of nest size and cotton ball use in *Uhrf2* knockout and littermates. (**A**) Observational comparison of behavioral patterns between wild-type mice (littermates; left box) and *Uhrf2* knockout mice (adjacent box) in the nest-building experiment. To calculate the percentage of cotton balls in the nest, we divided the number of grid cells containing cotton balls by the total number of cotton balls (12) and multiplied the result by 100. (**B**) Nest size of *Uhrf2* knockout mice (red, *n* = 10) was significantly larger than that of wild-type mice (gray, *n* = 8) at 1, 3, 5, 10, and 24 h time points. Statistical significance of the differences was determined using Sidak’s multiple comparisons test, with *p*-values for each time point as follows: at 1 h, *** *p* = 0.0004; at 3 h, *** *p* = 0.0006; at 5 h, * *p* = 0.036; at 10 h, ** *p* = 0.002; at 24 h, **** *p* < 0.0001. (**C**) The number of intact cotton balls remaining in the cages of *Uhrf2* knockout mice was significantly higher than in the cages of wild-type mice at 1, 3, and 5 h. The *p*-values obtained from the post hoc test were as follows: at 1 h, * *p* = 0.025; at 3 h, ** *p* = 0.003; at 5 h, ** *p* = 0.002; at 10 and 24 h, ns (not significant).

In our study, we evaluated the nesting behavior of *Uhrf2* knockout and wild-type mice by distributing cotton balls in a 12-cell grid overlay and analyzing the arrangement and collection of the cotton balls at different time points (Figure 1A). Analysis of nest size revealed a significant difference between the two groups at multiple time points, 1, 3, 5, 10, and 24 h. Specifically, *Uhrf2* knockout mice had a significantly larger nest size compared to wild-type mice (*p* < 0.0001). This finding was further supported by a two-way RM ANOVA, which revealed a significant effect of genotype (*F* (1, 16) = 41.25). Importantly, the lack of a significant interaction between time and genotype (*F* (4, 64) = 1.35, *p* = 0.3) indicated that the effect of genotype on nest size remained consistent across time points (Figure 1B).

In addition, our study examined the disassembly of cotton balls, providing valuable insight into the preparation of nest-building material. Analysis of the data in Figure 1C showed that *Uhrf2* knockout mice had a significantly higher number of intact cotton balls at multiple time points, indicating a decreased ability to disassemble them. A two-way RM ANOVA confirmed a significant genotype effect on the number of intact cotton balls (*F* (1, 16) = 8.54, *p* = 0.01). Sidak’s multiple comparisons tests also revealed significant group differences at 1, 3, and 5 h (see Figure 1 legend for specific values). These results provide valuable insight into the effects of *Uhrf2* gene deletion on nesting behavior in mice. In addition, they suggest adverse effects on temperature regulation and imply an overall decrease in nesting ability in *Uhrf2* knockout mice.

**Figure 2:**
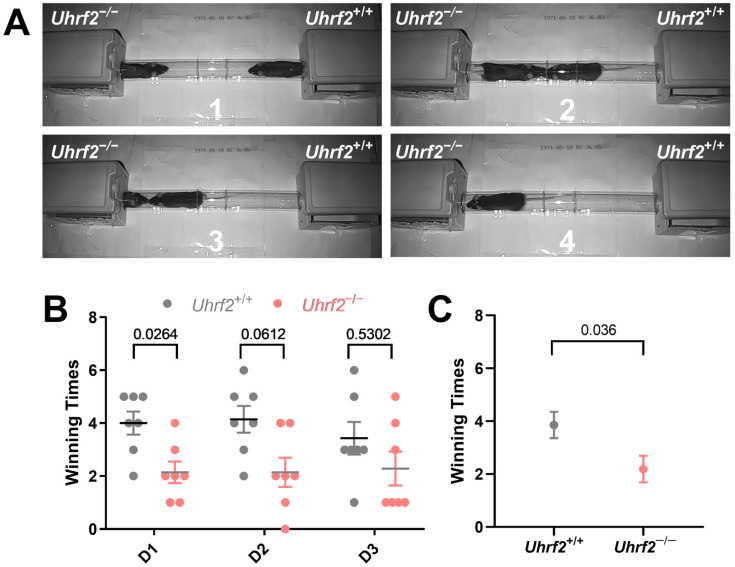
*Uhrf2* gene deletion impairs tube dominance behavior. (**A**) Observational comparison of behavioral patterns between *Uhrf2* knockout mice (left panel) and wild-type mice (right panel) in the tube dominance test (1 to 4: time sequence of wild-type mice winning). (**B**) The bar graph presents individual winning times of mice per day, as well as the average and standard error of the mean (SEM), illustrating the tube dominance test results for wild-type mice (gray, *n* = 7) and *Uhrf2* knockout mice (red, *n* = 7). On day one, *Uhrf2* knockout mice had significantly lower winning times than the wild-type mice. The values of Sidak’s test for multiple comparisons are shown above the brackets in the graph. (**C**) *Uhrf2* knockout mice exhibited significantly reduced average winning times across all days compared to their wild-type littermates (unpaired *t*-test, *t* = 2.35, df = 12, *p* < 0.05).

In addition to evaluating nest-building ability, we investigated the impact of *Uhrf2* knockout on broader social behaviors, particularly the establishment of dominance hierarchies. For this purpose, we conducted the tube test and observed that *Uhrf2* knockout mice exhibited a significant decrease in tube dominance compared to wild-type controls (Figure 2A,B). The outcomes of a two-way RM ANOVA elucidated a notable effect of genotype on winning times in the dominance tube test (*F* (1, 12) = 5.543, *p* = 0.036). However, the day of testing did not have a significant impact on the observed results (*F* (1.88, 22.56) = 0.87, not significant). Remarkably, *Uhrf2* knockout mice consistently displayed lower winning scores on the first day of testing and throughout the two consecutive days (Figure 2C). These findings indicate that *Uhrf2* knockout leads to diminished social dominance in comparison to littermates, potentially attributed to reduced aggression or heightened social anxiety.

**Figure 3:**
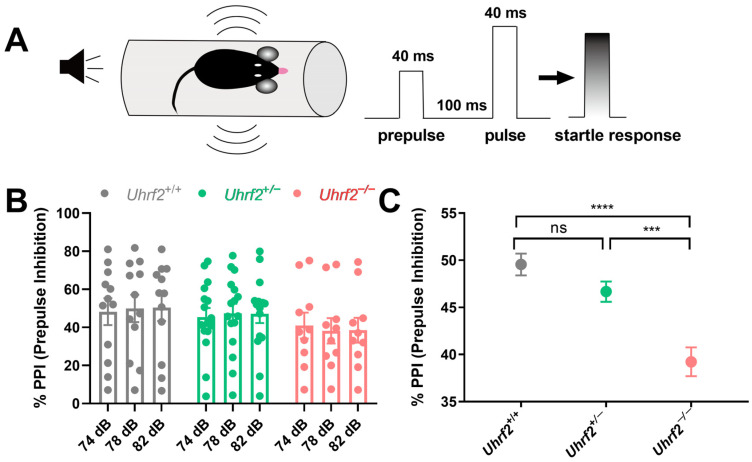
*Uhrf2* gene deletion results in impaired sensorimotor gating as measured by prepulse inhibition test (PPI). (**A**) A schematic of the PPI setup, the sequence of acoustic stimuli (S1 and S2), and the startle response measurement are presented. (**B**) Results of the PPI are shown for wild-type mice (gray, *n* = 12), heterozygous mice (green, *n* = 12), and *Uhrf2* knockout mice (red, *n* = 10) at sound pressure levels of 74, 78, and 82 dB. (**C**) The results show a significant difference in PPI between *Uhrf2*^+/+^ and *Uhrf2*^+/−^ mice compared to *Uhrf2*^−/−^ mice. Sidak’s multiple comparisons yielded the following values: **** *p* < 0.0001, *** *p* = 0.0004, and ns (0.07), respectively.

In addition to investigating social dominance, we explored the effects of *Uhrf2* knockout on various components of social communication, including PPI. Impaired PPI has been implicated in several neurodegenerative and psychiatric disorders, highlighting its importance in cognitive and behavioral domains [39]. PPI is based on the principle that the startle response to a loud acoustic stimulus (e.g., 120 dB) is modulated by the presence of a preceding moderately intense acoustic stimulus (Figure 3A). Although the one-way RM ANOVA analysis did not reveal any significant effect of the three different prepulse acoustic stimuli within each of the three groups on the startle response (*Uhrf2*^+/+^: *F* (1.96, 21.52) = 0.69, *p* = 0.51; *Uhrf2*^+/−^: *F* (1.99, 21.92) = 0.075, *p* = 0.93; *Uhrf2*^−/−^: *F* (1.85, 16.63) = 0.77, *p* = 0.47), the consistent variation observed across subjects suggests that the experimental conditions had a consistent effect across stimuli, regardless of the specific prepulse stimulus intensity used (Figure 3B). In addition, our results showed a significant effect of genotype on sensorimotor gating, as revealed by a one-way ANOVA analysis (*F* (2, 6) = 68.58, *p* < 0.0001). Specifically, *Uhrf2*^−/−^ mice displayed a deficit in PPI compared to their heterozygous and wild-type littermates, as evidenced by a significantly lower percentage of observed PPIs (Figure 3C). These results provide further insight into the multiple roles of *Uhrf2* in regulating different facets of social communication.

In summary, the results revealed significant behavioral differences between *Uhrf2* knockout mice and their wild-type littermates, suggesting a critical role for *Uhrf2* in the regulation of social behaviors. The knockout mice exhibited impairments in nest-building ability, reduced dominance levels in the dominance tube test, and suppressed pre-pulse inhibition. These findings suggest that *Uhrf2* and the gene expression network under its control are involved in the regulation of social behaviors, although the exact mechanisms and brain areas underlying these complex behaviors are not yet fully understood. Nonetheless, our results demonstrate that a deficiency in *Uhrf2* leads to changes in social behaviors, highlighting its importance in maintaining normal social interactions.

### 2.2. Social Memory of Uhrf2 Knockout Mice

In addition to assessing general social behaviors, we aimed to evaluate the impact of *Uhrf2* deficiency on short-term social memory, which is critically dependent on the CA2 region of the hippocampus. To achieve this, we conducted the three-chamber social interaction test, which enables the assessment of sociability and social memory in rodents [40].

**Figure 4:**
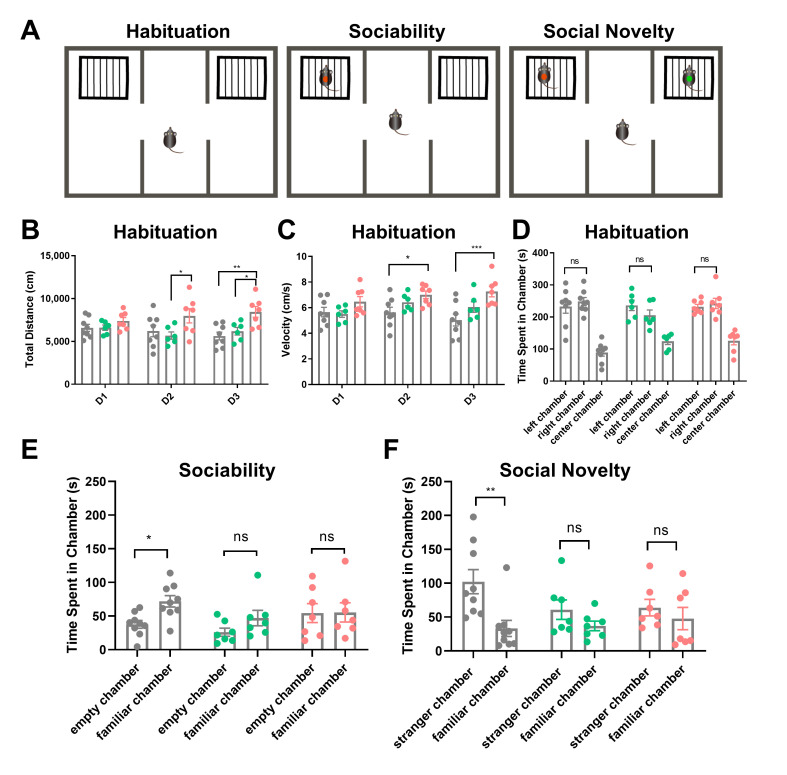
Analysis of social behavior in wild-type, heterozygous, and homozygous *Uhrf2* knockout mice using the three-chamber sociability and social novelty test. (**A**) The figure illustrates a schematic representation of the three-chamber social test. (**B**) The total distance traveled by mice with different genotypes was measured during the habituation period. A two-way RM ANOVA revealed a significant effect of genotype (*F* (2, 18) = 6.33, ** *p* = 0.008), but no significant effect of test day (*F* (2, 36) = 0.21, *p* = 0.82, ns). On the first day (D1), there was no significant difference in distance traveled among the three genotypes: wild-type mice (*Uhrf2*^+/+^, gray, *n* = 8), heterozygous mice (*Uhrf2*^+/−^, green, *n* = 6), and knockout mice (*Uhrf2*^−/−^, red, *n* = 7). However, on day 2 (D2), the heterozygous mice moved significantly shorter distances compared to the knockout mice (* *p* = 0.017). On day 3 (D3), the homozygous knockout mice traveled significantly longer distances than both the wild-type and heterozygous mice (** *p* = 0.001, * *p* = 0.02). (**C**) The velocity of mice with different genotypes was measured during the habituation period. Statistical analysis using a two-way RM ANOVA revealed a significant effect of genotype (*F* (2, 18) = 7.45, *p* = 0.004), while no significant effect of test days was observed (*F* (2, 36) = 1.59, *p* = 0.21; ns). On days 2 and 3, knockout mice traveled at a significantly higher velocity than wild-type mice (* *p* = 0.036, *** *p* < 0.0003). (**D**) The exploration preference of the mice during the habituation period on the test day (D4) was evaluated. A two-way RM ANOVA showed no significant effect of genotype (*F* (2, 18) = 3.37, *p* = 0.06, ns) and no significant effect of chamber (*F* (1, 18) = 0.01, ns) on the outcome of the test. There were no significant differences in exploration time between wild-type, heterozygous, and knockout mice in the left and right chambers (ns, not significant). (**E**) Sociability was assessed by measuring the amount of time mice spent exploring the familiar mouse compared to the empty cage. Wild-type mice spent significantly more time exploring the familiar mouse than the empty cage (* *p* = 0.043). However, no significant difference was observed between the exploration of the familiar mouse and the empty chamber in heterozygous and knockout mice (ns, not significant). A two-way repeated measures ANOVA revealed that the effect of genotype was not significant (*F* (2, 18) = 2.07, *p* = 0.16, ns), but the effect of chamber was significant (*F* (1, 18) = 5.16, *p* = 0.0357). (**F**) In terms of social novelty in mice, wild-type mice exhibited significantly higher levels of exploration towards the unfamiliar mouse compared to the familiar mouse (** *p* = 0.0025). Furthermore, no significant difference in exploration time between the unfamiliar and familiar mice was observed in heterozygous and knockout mice. A two-way repeated measures ANOVA revealed an effect of the chamber on the variations (*F* (1, 18) = 11.68, *p* = 0.003), but no significant effect of genotype (*F* (2, 18) = 0.94, *p* = 0.41). The *p*-values presented in this legend represent the results of comparisons analyzed using Sidak’s multiple comparisons.

During the three-day habituation phase, we measured the total distance traveled and velocity within the chambers. No significant differences were observed between wild-type littermates (*n* = 8) and *Uhrf2* knockout mice (*n* = 7) on the first day. However, on the second day of habituation, homozygous knockouts exhibited increased distance traveled compared to heterozygotes (*n* = 6, *p* = 0.016), along with higher velocity compared to wild types (*p* = 0.032). Furthermore, on the last day of habituation, *Uhrf2*^−/−^ mice exhibited greater distance and velocity compared to both heterozygous and wild-type littermates (*p* = 0.0011 and *p* = 0.018 for distance; *p* = 0.0003 for velocity) (see Figure 4B,C). These findings suggest that *Uhrf2*^−/−^ mice display a level of hyperactivity in the habituated context.

On the fourth day, we performed sociability and social novelty tests after 10 min of habituation, now with empty cages placed in the side chambers. No group differences arose in the exploration of the novel empty cages (Figure 4D). For sociability assessments, a familiar mouse was introduced into one cage and the exploration time was quantified. The wild-type mice exhibited greater exploration of the mouse cage compared to the empty cage (*p* = 0.043). However, *Uhrf2* heterozygous and homozygous knockouts showed no preference between the two cages (Figure 4E). Notably, our social approach assay differed from the standard protocol by utilizing a familiar mouse rather than an unfamiliar stranger mouse, thereby assessing not just sociability but also the integration of social memory.

In the subsequent social novelty test, wild-type mice explored the cage with the unfamiliar mouse significantly more than the cage with the familiar mouse. Heterozygotes showed a non-significant increase in exploration of the novel mouse. Notably, *Uhrf2*^−/−^ mice exhibited no difference in exploration between the unfamiliar and familiar cages (see Figure 4F).

In summary, the adapted protocol provides insight into social memory by quantifying interaction with a familiar mouse, while traditional sociability assesses preference for a stranger mouse. The results of tests assessing social memory showed that knockout mice performed significantly worse than wild-type littermates. Additionally, *Uhrf2* knockout mice exhibited impaired habituation over repeated exposures. After the initial habituation period, the knockouts displayed hyperactive locomotion compared to the wild types.

### 2.3. Characterization of Afferent Pathway-Specific Synaptic Plasticity in Mice with Different Genotypes

The hippocampal CA2 region has emerged as an integral locus for social cognition, including roles in social memory, recognition, and preference. Using immunofluorescence against RGS14, a marker protein for CA2 [7,41], the relative position within a transversal hippocampal slice could be depicted. To ascertain if *Uhrf2* knockout could perturb CA2-dependent processes, we performed in vitro electrophysiology to evaluate pathway-specific synaptic plasticity.

**Figure 5:**
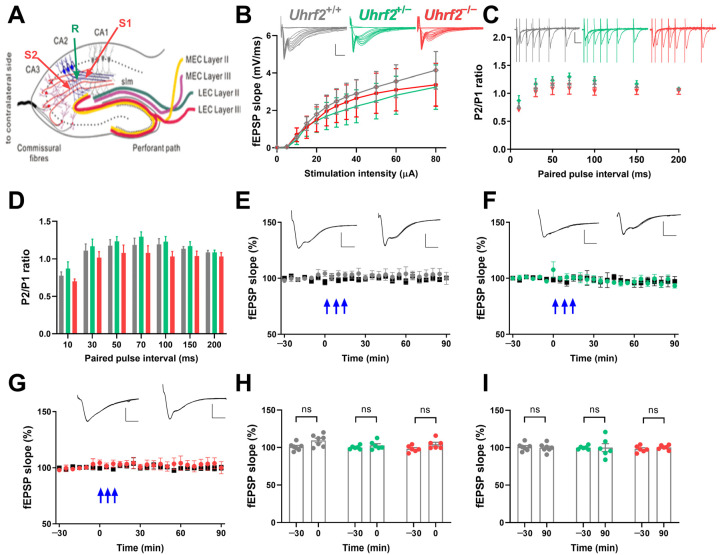
*Uhrf2* knockout does not affect input–output characteristics, paired-pulse facilitation, and synaptic plasticity in the SC-CA2 pathway. (**A**) This is a schematic diagram that illustrates the placement of electrodes in the SC-CA2 region. (**B**) Input–output curves were generated for the SC-CA2 synaptic transmission in *Uhrf2*^+/+^ (gray, *n* = 7), *Uhrf2*^+/−^ (green, *n* = 6), and *Uhrf2*^−/−^ mice (red, *n* = 7). fEPSP sample traces were presented with scale bars indicating an amplitude of 2 mV and a time interval of 5 ms. Statistical analysis using a two-way RM ANOVA revealed no significant differences in input–output curves among the different groups of mice (*F* (2, 17) = 0.13, *p* = 0.88). Post hoc comparisons using Sidak’s test also did not show any significant differences between groups per stimulation intensity (all *p*-values > 0.7). (**C**) Paired pulse facilitation fEPSP traces and ratios of P2/P1 fEPSP slopes were recorded for *Uhrf2*^+/+^ (gray, *n* = 8), *Uhrf2*^+/−^ (green, *n* = 6), and *Uhrf2*^−/−^ (red, *n* = 7) mice. Scale bars are 1 mV/10 ms. (**D**) No significant differences in paired-pulse facilitation were observed between *Uhrf2*^+/+^, *Uhrf2*^+/−^, and *Uhrf2*^−/−^ mice (two-way RM ANOVA: *F* (2, 18) = 1.78, *p* = 0.12). Post hoc group comparisons per stimulus interval also revealed no significant differences (all *p*-values > 0.1). (**E**) LTP induction was observed in wild-type *Uhrf2*^+/+^ mice (gray, *n* = 7) following three tetanic stimulations (3 × 100 Hz, indicated by blue arrows). (**F**) LTP induction was performed in *Uhrf2*^+/−^ mice (green, *n* = 6) after three tetanic stimulations. (**G**) LTP induction was also performed in *Uhrf2*^−/−^ mice (red, *n* = 6). (**H**) No detectable effect of genotype on fEPSP slope potentiation immediately after tetanization was observed in *Uhrf2*^+/+^, *Uhrf2*^+/−^, and *Uhrf2*^−/−^ mice (two-way RM ANOVA: *F* (2, 16) = 2.29, *p* = 0.14). Post hoc comparisons showed a significant difference between baseline and time 0 in wild-type mice (*p* = 0.044), while no significant differences were detectable in heterozygous and homozygous mice. (**I**) At the 90 min time point, there was no genotype effect on fEPSPs compared to baseline in *Uhrf2*^+/+^, *Uhrf2*^+/−^, and *Uhrf2*^−/−^ mice (two-way RM ANOVA: *F* (2, 16) = 0.12, *p* = 0.89). Post hoc comparisons also showed no significant differences between baseline and 90 min time points for any group (all *p*-values > 0.9). Representative fEPSP traces at −30 and 90 min are shown with 2 mV/5 ms scale bars.

First, we examined basal synaptic transmission and input–output relationship at Schaffer collateral (SC) to CA2 synapses across wild-type, heterozygous, and *Uhrf2* knockout mice. No significant differences arose in SC-CA2 synaptic transmission efficiency or field postsynaptic potential (fEPSP) input–output curves between groups (Figure 5A,B). Moreover, SC-CA2 paired-pulse facilitation was unaffected by *Uhrf2* deletion (Figure 5C,D).

We examined the induction of long-term potentiation (LTP) using 3 × 100 Hz stimulus trains and found no differences in SC-CA2 fEPSP potentiation between genotypes (Figure 5E–I). In addition, we performed a two-way RM ANOVA to directly compare the time course of the fEPSP slope after tetanization among the three groups (Figure 5E–G). The analysis revealed no significant effect of genotype on fEPSP slope over time (*F* (2, 16) = 0.61, *p* = 0.56), and no significant effect of time (*F* (2.94, 47.04) = 2.63, *p* = 0.062) on fEPSP values. These results support previous studies indicating that SC-CA2 synapses lack activity-dependent plasticity [15,16,18].

Previous studies have shown that synapses form between entorhinal cortex (EC) afferents and CA2 neurons exhibit long-term potentiation (LTP) similar to Schaffer collateral–CA1 synapses [15]. Furthermore, reducing the activity of the entorhinal cortex afferents to the hippocampus decreased the extent of memory formation for new social encounters, highlighting their significance in enduring social interactions.

**Figure 6:**
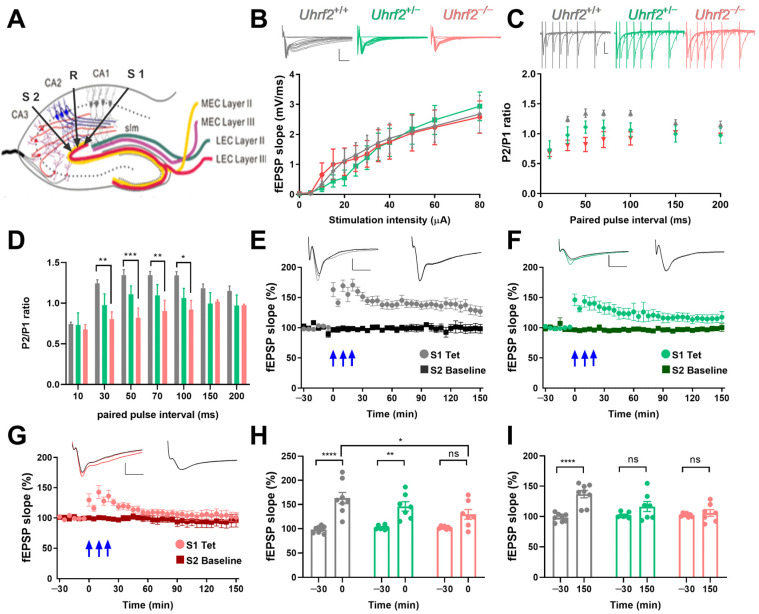
*Uhrf2* knockout impairs paired-pulse facilitation and LTP at EC-CA2 synapses. (**A**) Schematic showing electrode placement in the EC-CA2 pathway. (**B**) Input–output curves show the dynamics of the fEPSP slope in response to stimulation intensity (0–80 µA) for *Uhrf2*^+/+^ (gray, *n* = 10), *Uhrf2*^+/−^ (green, *n* = 6), and *Uhrf2*^−/−^ mice (red, *n* = 7). The input–output curves show no significant differences among the three groups of mice (two-way RM ANOVA: *F* (2, 20) = 0.025, *p* = 0.97). Scale bars: 2 mV/5 ms. (**C**) fEPSP traces recorded in response to paired-pulse facilitation stimulation using 40% of the maximum slope as the stimulation intensity in *Uhrf2*^+/+^ (gray, *n* = 8), *Uhrf2*^+/−^ (green, *n* = 6), and *Uhrf2*^−/−^ mice (red, *n* = 7). The scale bar is 1 mV/10 ms. (**D**) At 30, 50, 70, and 100 ms intervals, paired-pulse facilitation was significantly lower in *Uhrf2* knockout mice compared to wild-type mice. A two-way RM ANOVA revealed a significant effect of genotype (*F* (2, 18) = 4.70, *p* = 0.021). Post hoc Sidak’s tests for paired-pulse intervals of 30 ms (** *p* = 0.0090), 50 ms (*** *p* = 0.0007), 70 ms (** *p* = 0.008), and 100 ms (* *p* = 0.015) showed significant differences between knockout and wild-type mice. (**E**) Induction of LTP was also recorded in hippocampal slices from *Uhrf2*^+/+^ mice (gray, *n* = 8) using three tetanic stimulations (3 × 100 Hz, indicated by blue arrows). (**F**) Induction of LTP was recorded in *Uhrf2*^+/−^ mice (green, *n* = 7). (**G**) The induction of LTP was also observed in *Uhrf2* knockout mice (red, *n* = 7). Scale bars: 1 mV/5 ms. (**H**) The fEPSP slope in EC-CA2 increased significantly after tetanization (0 min) in wild-type and heterozygous mice, whereas no significant difference was observed in knockout mice compared to baseline. The effect of genotype at this time point was not significant (*F* (2, 19) = 1.77, *p* = 0.20, ns). However, baseline to first value after tetanization was significant for wild-type mice (**** *p* < 0.0001) and heterozygous mice (** *p* = 0.002) but not for homozygous knockout mice (ns, *p* = 0.06). Additionally, the first value after tetanization in wild-type mice differed significantly from that in knockout mice (* *p* = 0.011). (**I**) There was a significant effect of genotype on fEPSP potentiation at 150 min after tetanization, based on a two-way RM ANOVA (*F* (2, 19) = 4.5, *p* = 0.025). In addition, fEPSP potentiation was significantly higher than baseline only in wild-type mice (**** *p* < 0.0001) but not in recordings from heterozygous or homozygous knockout mice. There were also significant differences between wild-type and heterozygous mice (*** *p* = 0.0002) and between wild-type and homozygous knockout mice (* *p* = 0.016). Brackets and asterisks in the graphs represent Sidak’s multiple comparison *p*-values: **** *p* < 0.0001, ** *p* < 0.002, and ns (not significant). The figure legend also provides the specific *p*-values of the post hoc tests for comparisons.

Therefore, we investigated the impact of *Uhrf2* knockout on EC-CA2 plasticity. Although input–output curves were similar across all groups (Figure 6A,B), EC-CA2 paired-pulse facilitation was significantly reduced in *Uhrf2*^−/−^ mice (Figure 6C,D). This implies that *Uhrf2* selectively influences short-term plasticity dynamics at EC-CA2 synapses.

Next, we investigated the long-term potentiation (LTP) in the EC-CA2 pathway. To directly compare the time course of the fEPSP slope after tetanization over 150 min among the three groups, we examined the data as shown in Figure 6E–G. The comparison was made using a two-way RM ANOVA, which revealed a significant effect of genotype on the fEPSP slope over time (*F* (2, 19) = 6.377, *p* = 0.0076).

A statistical comparison of the fEPSP slopes at different time points revealed that immediately after tetanization, both wild-type and heterozygous mice showed a significant increase in the slope compared to the baseline. However, in knockout mice, there was no significant difference between the fEPSP slopes of the tetanized synaptic input and the control input (Figure 6H). At the 150 min mark, the fEPSP slope in wild-type mice remained significantly higher than the baseline. In contrast, the slopes of heterozygous and knockout mice returned to baseline levels (Figure 6I).

The different response profiles of the fEPSP slope over time demonstrate how genetic variations influence synaptic plasticity. These results, depicted in Figure 6H,I, underscore the significance of genotype in shaping the dynamics of fEPSP slope potentiation and provide insights into the physiological implications of the observed genetic variation.

Taken together, these results suggest *Uhrf2* knockout spares SC-CA2 synaptic transmission and plasticity, while selectively disrupting plasticity processes localized to the EC-CA2 pathway. These results shed light on the role of *Uhrf2* in the hippocampus and suggest that *Uhrf2* may be involved in the regulation of synaptic plasticity and information processing in the brain. Further elucidation of the molecular underpinnings of this effect could have important implications for comprehension of *Uhrf2*-mediated regulation of hippocampal learning and memory.

### 2.4. Transcriptomic Analysis Reveals Altered Gene Expression in the Hippocampus of Uhrf2 Knockout Mice

**Figure 7:**
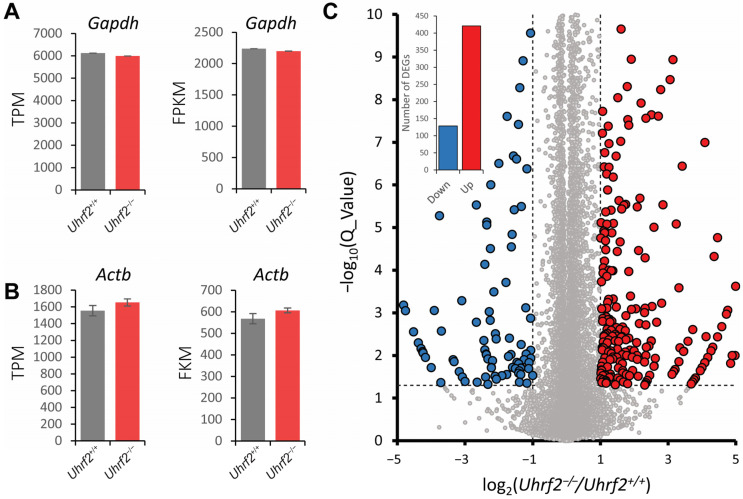
Identification of differentially expressed genes (DEGs) in the hippocampus of *Uhrf2* knockout mice using RNA sequencing. (**A**) The TPM (transcripts per million) and FPKM (fragments per kilobase million) expression of the housekeeping gene *Gapdh* in *Uhrf2*^+/+^ (gray, *n* = 3) and *Uhrf2*^−/−^ (red, *n* = 3) mice did not show a significant difference. (**B**) Similarly, the TPM and FPKM expression of the housekeeping gene *Actb* in *Uhrf2*^+/+^ (gray, *n* = 3) and *Uhrf2*^−/−^ mice (red, *n* = 3) show no significant difference. (**C**) The volcano plot displays the distribution of DEGs caused by *Uhrf2* knockout, with upregulated genes in red and downregulated genes in blue. Only genes with Q values < 0.05 (−log10(0.05) = 1.3, horizontal dashed line) and log2(*Uhrf2*^−/−^/*Uhrf2*^+/+^) < −1 or > 1 (vertical dashed line) were considered DEGs. Genes that did not meet these criteria are shown as gray circles. The inset bar graph shows the total number of downregulated and upregulated DEGs associated with *Uhrf2* knockout. Notably, some of these DEGs have established links to cognition. For instance, *Gnb3* encodes a G protein subunit implicated in neurotransmitter signaling and risk for neurological disorders. Studies have shown that the *Gnb3* gene is associated with the onset of neurological disorders such as depression and dementia.

**Figure 8:**
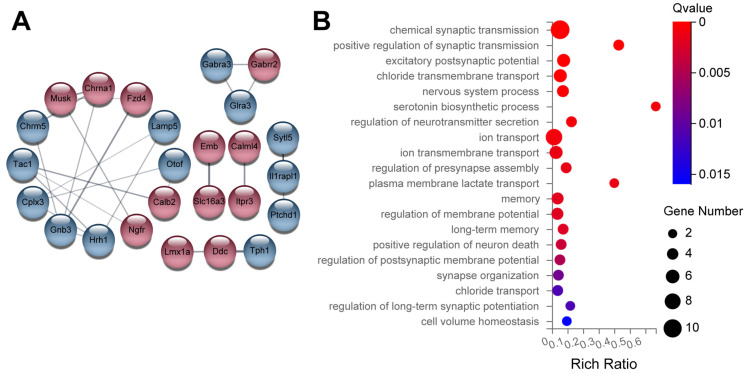
Examples of DEGs related to synaptic transmission and regulation of synaptic plasticity. (**A**) The diagram shows genes that are DEGs and associated with synaptic transmission and regulation of synaptic plasticity. Red circles represent upregulated genes, while blue circles indicate downregulated genes. Notable highly integrated genes include *Gnb3* and *Hrh1*. The numbers in (**B**) represent the number of DEGs in each enriched category, as represented by the size of the Gene Ontology pathway (GO pathway) circle. The Q values are color-coded to display the ratio of identified DEGs to the total number of genes in the category index. The DEGs are primarily associated with chemical synaptic transmission, excitatory postsynaptic potential, chloride ion transport, vesicle transport, and regulation of membrane potential.

To gain insight into potential molecular mechanisms underlying the influence of *Uhrf2* on cognition and synaptic function, we performed RNA sequencing on hippocampal tissue from *Uhrf2* knockouts and wild-type littermates. Subsequent bioinformatic analysis identified differentially expressed genes (DEGs) and affected pathways resulting from *Uhrf2* deletion. As expected, *Uhrf2* exon 1 expression was absent in knockouts, as confirmed by analysis of reads from the RNA-seq data (Supplemental Figure S2). Expression of the housekeeping genes *Gapdh* and *Actb* remained unchanged (Figure 7A,B). Numerous DEGs arose from *Uhrf2* knockout, including 421 upregulated and 129 downregulated genes (Figure 7C). Further examination highlighted DEGs involved in synaptic transmission and plasticity, such as *Gnb3*, *Hrh1*, *Gabra2*, and *Gabra3* (Figure 8A,B).

Some of the DEGs have established links to cognition. For example, *Gnb3* encodes a G protein subunit involved in neurotransmitter signaling and has been associated with an increased risk of neurological disorders [42]. Studies have shown that the *Gnb3* gene is related to the occurrence of neurological diseases, such as depression and dementia [43].

The histamine receptor *Hrh1* plays a role in memory, and its deletion disrupts hippocampal plasticity [44,45]. *Gabra2* and *Gabra3* encode subunits of the GABAA receptor, which is the primary receptor for the GABA neurotransmitter in the brain. GABA is the main inhibitory neurotransmitter in the central nervous system of mammals. GABAA receptors, along with excitatory glutamate, play a crucial role in maintaining the balance between inhibitory and excitatory responses, which is essential for the normal functioning of the brain. When activated, GABAA receptors selectively transmit chloride ions, leading to the hyperpolarization of neurons and regulating synaptic plasticity [46,47].

RNA-sequencing analysis provides novel insights into the molecular mechanisms underlying the effect of *Uhrf2* knockout on synaptic transmission and plasticity. The identification of specific DEGs related to synaptic plasticity and neurotransmission opens new avenues for further investigation into the role of *Uhrf2* in hippocampal function and its potential relevance to neurological diseases.

## 3. Discussion

Our findings elucidate the multiple roles of *Uhrf2* in modulating social behavior and memory processes in mice. Behavioral assays revealed that *Uhrf2* deficiency results in deficits in multiple domains of social interaction, including nest-building ability, dominance hierarchy establishment, and sensorimotor gating as measured by PPI. In addition, assessments relying on intact short-term social memory, mediated in part by entorhinal cortex-to-hippocampal CA2 neuronal circuits, revealed additional deficits resulting from *Uhrf2* deletion. Electrophysiological analyses supported aberrant synaptic plasticity specific to the entorhinal cortex–CA2 pathway as a potential mechanism linking *Uhrf2* to the regulation of social cognition. However, RNA sequencing revealed that *Uhrf2* knockout also affected broad gene expression networks involving vesicle trafficking, transcriptional regulation, and neuroepithelial cell differentiation pathways. This suggests that the collective effects of *Uhrf2* loss on social behavior, memory, and synaptic plasticity likely result from multiple effects on molecular processes beyond a single mechanism.

### 3.1. Uhrf2 Deletion Results in Reduced Nest Building Speed, Tube Dominance, and Prepulse Inhibition

Our studies revealed that *Uhrf2* deletion results in reduced nest-building ability, social dominance, and sensorimotor gating quantified by the prepulse inhibition test in mice. Nest building facilitates thermoregulation, reproduction, and shelter construction in mammals, and deficiencies are associated with brain dysfunction.

Specifically, we found that *Uhrf2*-deficient mice had a reduced ability to disassemble cotton balls at different intervals and built a larger nest with the same number of cotton balls, suggesting reduced nesting behavior. While some research suggests that disruption of the UPS could disrupt nesting behavior, the links between *Uhrf2* and nesting ability remain unexplored. Similarly, despite reduced dominance in the tube test in *Uhrf2* knockouts, clear links between *Uhrf2* and dominance have yet to emerge. Of note, proper cognitive and behavioral function relies on complex, interconnected brain networks rather than isolated regions. The broad behavioral perturbations observed following *Uhrf2* knockout may result from diverse effects on neural circuits rather than singular mechanisms. *Uhrf2* deletion also attenuated prepulse inhibition, which has been associated with neuropsychiatric conditions [48,49]. This is consistent with the spectrum of social behavioral deficits seen with *Uhrf2* loss. Taken together, our behavioral findings demonstrate that *Uhrf2* contributes broadly to the orchestration of social functioning in mice, potentially through effects on multiple neural pathways involved in these diverse behaviors. Elucidating the precise brain regions and molecular interactions that govern *Uhrf2′*s role is a goal for future mechanistic dissection.

### 3.2. Uhrf2 Deletion Impairs Social Novelty

In addition to general social behaviors, our studies revealed that *Uhrf2* knockout mice failed to habituate to the test apparatus over three repeated exposures. The knockouts also exhibited increased locomotor activity compared to wild-type controls during the habituation phase. Concerning short-term social memory, *Uhrf2* deletion significantly impaired performance compared to wild-type littermates. Interestingly, we did not find any previous publications explicitly linking *Uhrf2* loss to habituation, hyperlocomotion, or social memory deficits in mice. However, accumulating evidence highlights the hippocampal CA2 subregion as a critical site for social novelty and memory consolidation, with dysfunction potentially contributing to social impairments in several brain disorders [10]. In particular, lateral projections from the entorhinal cortex to CA2 represent a central circuit for stabilizing social memories, with plasticity in this pathway regulating social cognition [17,50]. Therefore, the potential changes in synaptic plasticity in *Uhrf2* knockout mice within the EC-CA2 circuit may be involved in the regulation of social memory.

### 3.3. Uhrf2 Deletion Attenuates LTP in EC-CA2 Synapse

While *Uhrf2* deletion spares SC-CA2 synaptic transmission and plasticity, our studies revealed a selective disruption of plasticity mechanisms localized to the entorhinal cortex–CA2 pathway. Specifically, electrophysiological studies revealed a selective disruption of LTP at EC-CA2 synapses in *Uhrf2* knockouts, while LTP at SC-CA2 synapses remained absent. We found that *Uhrf2* knockout did not alter SC-CA2 input–output relationships or paired-pulse facilitation, indicating that basal synaptic transmission was unaffected. However, *Uhrf2* loss significantly suppressed short-term plasticity dynamics measured by paired-pulse responses at EC-CA2 synapses, suggesting a deficit in presynaptic function. Critically, we observed a robust inhibition of both the induction and maintenance phases of long-term potentiation exclusively at EC-CA2 synapses in *Uhrf2* knockouts. This suggests a critical role for *Uhrf2* in enabling long-term synaptic strengthening, which is thought to underlie memory formation within the EC-CA2 circuit. While studies directly examining the involvement of ubiquitin pathways in EC-CA2 plasticity are limited, more general evidence suggests that UPS components can modulate synaptic plasticity and cognition through transcriptional and protein turnover mechanisms [51]. Therefore, our findings provide the first evidence implicating *Uhrf2* in the coordination of EC-CA2 plasticity and warrant further mechanistic studies to elucidate its potential role in enabling the long-lasting synaptic strengthening hypothesized to underlie memory formation within this circuit.

### 3.4. Changes of Gene Expression by Uhrf2 Knockout

*Uhrf2* knockout resulted in the upregulation of 421 genes and the downregulation of 129 genes. The results of the study, which used RNA sequencing technology, indicate that *Uhrf2* knockout leads to DEGs related to vesicle trafficking and transcription factors. Concerning vesicle trafficking, *Uhrf2* downregulation leads to a decrease in *Actc1* and *Myl2*, which encode actin and myosin, respectively. Actin is an essential structural protein that plays a critical role in cell functions such as division, migration, and vesicle trafficking [52]. Similarly, ACTC1 is one of the isoforms of myosin involved in early mammalian neurodevelopment, particularly in brain and glial cells (Goggolidou [53,54,55]. In addition, ACTC1 regulates vesicle trafficking and interacts with calcium–phospholipid-binding proteins [56,57]. The *Myl2* gene encodes myosin regulatory light chain, which specifically binds to myosin heavy chain, an essential component of myosin filaments. MYL2 is also involved in the AMPK pathway, which regulates energy balance in eukaryotes, and abnormal expression of MYL2 is associated with schizophrenia [58,59]. The study suggests that UHRF2 may regulate the transport and release of presynaptic vesicles by modulating ACTC1 and MYL2. The deletion of *Uhrf2* also affects the expression of genes related to neuroepithelium development. During early brain development, neuroepithelial cells interact with cerebrospinal fluid, which promotes brain expansion and development. The flow of cerebrospinal fluid is crucial for clearing metabolic waste from the brain, eliminating inflammatory factors, and regulating the physiological status of mice through neuroimmune responses [60,61,62,63,64]. *Six3*, which regulates neural development by interacting with signaling factors, is significant in mRNA sequencing results and has a significant increase in the content of SIX3 protein in *Uhrf2* knockout mice. Although there is no direct evidence that *Six3* is involved in learning and memory, studies have shown that *Six3* deficiency hinders the formation of dopamine receptor type 2-expressing striatal medium spiny neurons (D2-MSNs) that express dopamine receptor 2 (DRD2) in the striatum. Activation of D2-MSNs in the nucleus accumbens reduces cocaine-induced conditioned place preference memory [65,66,67,68].

We acknowledge that our study has several limitations. In particular, we did not examine the downstream functional consequences of differential expression of genes related to vesicle trafficking, transcription factors, or neuroepithelial cell differentiation. In addition, we did not directly examine the role of UHRF2-mediated transcriptional changes in memory processes, particularly in the CA2 area of the hippocampus. However, our integrative approach combining behavioral, electrophysiological, and genomic data provides novel evidence for the potential involvement of UHRF2 in modulating learning and memory capacities that support social cognition. Notably, UHRF2 appears to play a larger role in regulating hippocampal function through intricate effects on gene expression networks related to synaptic plasticity than previously recognized.

Moreover, the observed behavioral impairments in *Uhrf2* gene-deleted mice may involve contributions from motor and sensory alterations. Our RNA sequencing analysis revealed potential mechanisms related to GABA synthesis that affect the excitatory–inhibitory balance in neuronal inputs. Perturbations in this balance are associated with neurological disorders and changes in social behavior, such as those seen in schizophrenia. In addition, we found increased hyperactivity, suggesting motor dysfunction. However, pinpointing specific physiological mechanisms at the systemic level is challenging due to substantial changes in overall gene expression. Further studies are needed to understand the intricate relationship between gene expression changes, motor and sensory alterations, and the resulting behavioral phenotypes in *Uhrf2* knockout mice. Overall, our study highlights the multidimensional nature of the observed behavioral impairments in *Uhrf2* knockout mice.

Increased trimethylation of histone H3 lysine 9 in hematopoietic stem/progenitor cells in *Uhrf2*^−/−^ mice has been reported, indicating impaired repopulating ability and functioning of hematopoietic progenitors [69]. Additionally, *Uhrf2* accumulation has been observed in retinal progenitor cells (RPCs), and its conditional deletion resulted in reduced 5hmC levels, altered gene expressions, and disruption of retinal cell proliferation and differentiation [70]. Furthermore, *Uhrf2* has been implicated in the ubiquitination and degradation of nuclear aggregates containing polyglutamine repeats, such as seen in Huntington’s disease and related polyglutamine diseases [71]. These findings highlight the potential effects of *Uhrf2* beyond the hippocampus, encompassing alterations in hematopoietic cells, retinal progenitor cells, protein degradation processes, synaptic transmission, and transcriptional regulation.

## 4. Materials and Methods

### 4.1. Ethics Statement

We made efforts to minimize animal usage. All experiments adhered to the guidelines of the Institutes of Brain Science and State Key Laboratory of Medical Neurobiology of Fudan University, Shanghai, China, and were approved by the Institutional Animal Care and Use Committee of Fudan University, Shanghai Medical College (approval Nr. 31320103906).

### 4.2. Animals

To characterize the effects of genome-wide *Uhrf2* knockout, we utilized a mutant mouse strain with *Uhrf2* deletion generated via CRISPR-Cas9 targeting the start codon in exon 1. This mouse strain was previously created and validated by Dr. Dali Li’s group at East China Normal University [72,73]. Founder mice were kindly provided by Dr. Dali Li. Breeding colonies were maintained at the Institutes of Brain Science, Fudan University. Experimental animals included *Uhrf2*^−/−^ knockouts, as well as *Uhrf2*^+/−^ and *Uhrf2*^+/+^ littermate controls derived from *Uhrf2*^+/−^ x *Uhrf2*^+/−^ crosses. Genotyping utilized PCR with the forward primer CCATTTCTTGCTCACGCCAG and the reverse primer TCGGGCCTTACATCGAAGAG (Chen et al., 2017). Mice aged 6–8 months were group housed, with four mice per cage. They were kept in a 12 h reversed light/dark cycle at ambient temperature, with ad libitum access to food and water.

### 4.3. Observation of Nest-Building Behavior

To evaluate nest-building behavior, we distributed twelve 1 cm cotton balls evenly across the cage floor. The arrangement of the cotton balls within a 12-cell grid overlay was analyzed at different time points, following the methods described by Deacon, Jirk of et al., and Li et al. [37,74,75]. The analysis included assessing the amount of biting and shredding of the cotton balls, as well as their collection in a corner, within the 12-cell grid (Figure 1). To calculate the percentage of cotton balls in the nest, we divided the number of grid cells containing cotton balls by the total number of cotton balls (12) and multiplied the result by 100.

### 4.4. Social Tube Dominance Test

The tube test, also known as the social tube dominance test, is a widely used experimental paradigm to assess social hierarchies in mice [76,77]. A transparent tube was selected with a diameter that allowed forward and backward movement, enabling the evaluation of social hierarchies. During the habituation period, mice were given three consecutive days to freely explore the tube. On one of the competition days, when both mice entered the tube from opposite entrances, the dominant mouse displacing the other mouse scored one winning time (point), while the displaced mouse scored 0 points. To ensure fairness, we employed a round-robin design to semi-randomize pairings, guaranteeing that each wild-type (WT) mouse would compete against each knockout (KO) mouse. As a result, every mouse participated in a total of seven runs, with a new run beginning against a randomly selected competitor once all other mice in the group had finished their contests. Therefore, a maximum of seven wins per mouse per day could be achieved. However, there were instances where both mice remained in the tube, resulting in neither mouse receiving a victory point. This process was repeated over the next two days.

### 4.5. Prepulse Inhibition Test

Prepulse inhibition (PPI) is a well-established experimental paradigm used to study sensorimotor gating in rodents [49,78,79]. In this experiment, we induced and quantified PPI using acoustic stimuli and a mouse startle response recording system. The experiment included two types of acoustic stimuli, referred to as S1 and S2, which had different sound intensities. The PPI experiment consisted of eight trials, with each trial involving the presentation of S1 and S2 stimuli at a specific dB combination. Trial 2 served as a baseline measurement with S1 at 0 dB and S2 at 120 dB. Trials 4, 6, and 8 induced prepulse inhibition with S1 levels of 74, 78, and 82 dB, respectively, while S2 remained at a fixed intensity of 120 dB. In trials 3, 5, and 7, S2 was set to 0 dB and S1 was set to 74, 78, and 78 dB, respectively. These trials were designed to evaluate prepulse inhibition at different sound intensities by measuring the startle response of the mice. Each trial was randomly presented six times throughout the experiment. The PPI was calculated by comparing the mean startle response values of S2 in trials 4, 6, and 8 (averaging the values from the six repetitions of each trial) with the baseline value from trial 2. The percentage PPI for a given S1–S2 combination in trials 4, 6, and 8 was determined using the formula: %PPI = 100 − (mean of S2/mean of baseline) × 100%.

### 4.6. Three-Chamber Tasks: Sociability and Social Memory

We followed the previously published methods with minor modifications [40,80]. Briefly, we habituated the mice for three consecutive days by allowing them to move freely in the 3-chamber box for 20 min. The testing phase consisted of a final 10 min of habituation, a 10 min sociability test with a familiar mouse in one chamber, and a 10 min social novelty test with the familiar mouse in a cage of one chamber and an unfamiliar mouse in another cage in the opposite chamber. This study measured the extent of exploration and interaction with cages through video analysis using a tracking software package (EthoVision 14, Noldus (Beijing) Information Technology Co. Ltd., Beijing, China). The total time spent by the animals in the vicinity (within 2 cm) of the cages in either the left or right chamber was calculated by summing the respective time periods during which the animal’s nose tip was detected within the specified distance range.

### 4.7. Preparation of Acute Hippocampal Slices

The preparation of acute hippocampal slices followed previously published methodologies [26,75]. After anesthetizing the animal using isoflurane, the brain was removed immediately and immersed in ice-cold artificial cerebrospinal fluid (ACSF) composed of the following (in mM): 124 NaCl, 4.9 KCl, 1.2 KH_2_PO_4_, 25.6 NaHCO_3_, 1.3 MgSO_4_, 2.5 CaCl_2_, 10 d-glucose, and previously bubbled with 95% O_2_ and 5% CO_2_. Transverse slices (350 μm thick) were cut perpendicularly to the long axis of the hippocampus with a vibratome (VT-1200, Leica, Germany) and incubated for at least 2 h after slicing in a custom-made interface-type recording chamber at 32.5 °C under the constant perfusion with carbogenated ACSF at a flow rate of 4 mL/min.

### 4.8. Field Excitatory Postsynaptic Potentials (fEPSPs) Recording

The field excitatory postsynaptic potentials (fEPSPs) were evoked by stimulation with biphasic rectangular current pulses (100 μs/polarity) in a range of 15–25 μA through tungsten electrodes (A-M Systems) [18,75]. Stimulation was applied either at Schaffer collateral fibers for SC-CA2 recording or at axon terminals from the entorhinal cortex for EC-CA2 recording. The fEPSPs were recorded from the CA2 region using stainless steel electrodes (5 MΩ, A-M Systems) placed either in the stratum radiatum or stratum lacunosum–moleculare. The recording electrode (Rec) was positioned between the two independent stimulation inputs (S1 and S2) along the two different layers (Supplementary Figure S1A,B for location of CA2). The strength of synaptic transmission was determined by measuring the slope of the fEPSP. The stimulation intensity was set to 40–50% of the maximum fEPSP response. The size of fEPPSs was monitored by stimulation every 5 min as an average of four sweeps, with 10 s intervals between each sweep, throughout the experiment. Field potentials were recorded and digitized at a sampling frequency of 10 kHz using a CED 1401 plus AD/DA converter (Cambridge Electronics Design, Cambridge, UK). LTP was induced by a tetanization (TET) protocol consisting of three trains of 100 stimuli at 100 Hz separated by 10 min.

### 4.9. Immunofluorescence

For immunofluorescence, animals were anesthetized with 4% isoflurane and acute hippocampal slices were prepared as described in the section on slice preparation. The slices were then placed in 4% paraformaldehyde for 1 h at room temperature. After washing with PBS, the slice was transferred to a 30% sucrose solution in PBS and left overnight at 4 °C. Sections (40 μm) were cut with a cryotome (Leica Biosystems, Wetzlar, Germany). The sections were then mounted and permeabilized with 0.3% Triton X-100 in PBS for 30 min, and nonspecific binding sites were blocked with 5% goat serum in PBS for two hours at room temperature. Sections were then incubated with primary antibody (anti-RGS14 1:200; Abcam, Waltham, MA, USA) overnight at 4 °C. After three washes with PBS, sections were incubated with a species-specific secondary antibody (goat anti-rabbit Alexa 546, 1:400; Abcam) in 5% goat serum for 1 h. After application of DAPI in 0.01 M PBS (100 ng/mL, 28718-90-3, Roche, Switzerland) for 5 min and three additional washes with PBS, the sections were mounted onto slides (Fluoromount Aqueous Mounting Medium, Sigma, Livonia, MI, USA), and fluorescence images were captured using a fluorescence microscope system.

### 4.10. RNA Sequencing and Analysis

Total RNA was isolated from the hippocampi of *Uhrf2* knockout and wild-type littermate mice using TRIzol reagent (ThermoScientific, Shanghai, China) according to the manufacturer’s instructions. RNA sequencing was performed by the Beijing Genomics Institute. RNA sequencing libraries were prepared according to the Illumina TruSeq protocol v2 (Illumina, San Diego, CA, USA). Gene expression levels were determined using edgeR v3.32.1 software (https://bioconductor.org/) with detection rate adjustment and reported as fragments per kilobase of transcript per million mapped reads. A gene was considered to be expressed if it had fragments per kilobase per million greater than one. For a gene to be considered differentially expressed, it had to meet two criteria: a greater than 2.0-fold change in expression level and a *p*-value < 0.05 [81,82].

### 4.11. Statistical Analysis

Data are presented as mean ± standard error of the mean (SEM). Statistical analysis was performed using GraphPad Prism 8. For comparisons between more than two groups with a single independent variable, ANOVA was used. In the case of two independent variables, two-way RM ANOVA was used. Any additional statistical methods used are mentioned in the text. A *p*-value less than 0.05 was considered to indicate a statistically significant difference between groups or an effect on independent variables. The significance values of the tests performed are given in the text of the figure legends and/or in the graphs themselves.

## 5. Conclusions

In summary, our behavioral, electrophysiological, and transcriptomic findings implicate UHRF2 in the regulation of social interaction, memory, and synaptic potentiation in the entorhinal cortex–CA2 pathway. While further research is imperative to elucidate the precise mechanisms involved, this study provides evidence that UHRF2 likely contributes to the modulation of learning, memory, and goal-directed behaviors related to social functioning through coordinated effects on multiple molecular pathways. Targeted investigation of these complex gene regulatory networks mediated by UHRF2 may provide novel insights into neurological disorders characterized by social dysfunction.

## Data Availability

The original contributions presented in the study are included in the article/supplementary files; further inquiries can be directed to the corresponding author. The original RNA-sequencing data are available in SRA and accessible under the link: https://dataview.ncbi.nlm.nih.gov/object/PRJNA1044912.

## Supplementary Materials

The supporting information can be downloaded at: https://www.mdpi.com/article/10.3390/ijms25031543/s1.
