# Supplementary material for: E3 Ubiquitin Ligase Uhrf2 Knockout Reveals a Critical Role in Social Behavior and Synaptic Plasticity in the Hippocampus
Source: Int J Mol Sci. 2024 Jan 26;25(3):1543. doi: 10.3390/ijms25031543 (PMC10855348; doi:10.3390/ijms25031543)
Supplement: Supplementary file 1 [file ijms-25-01543-s001.zip › ijms-2766719-supplementary.pdf]

Supplemental Data

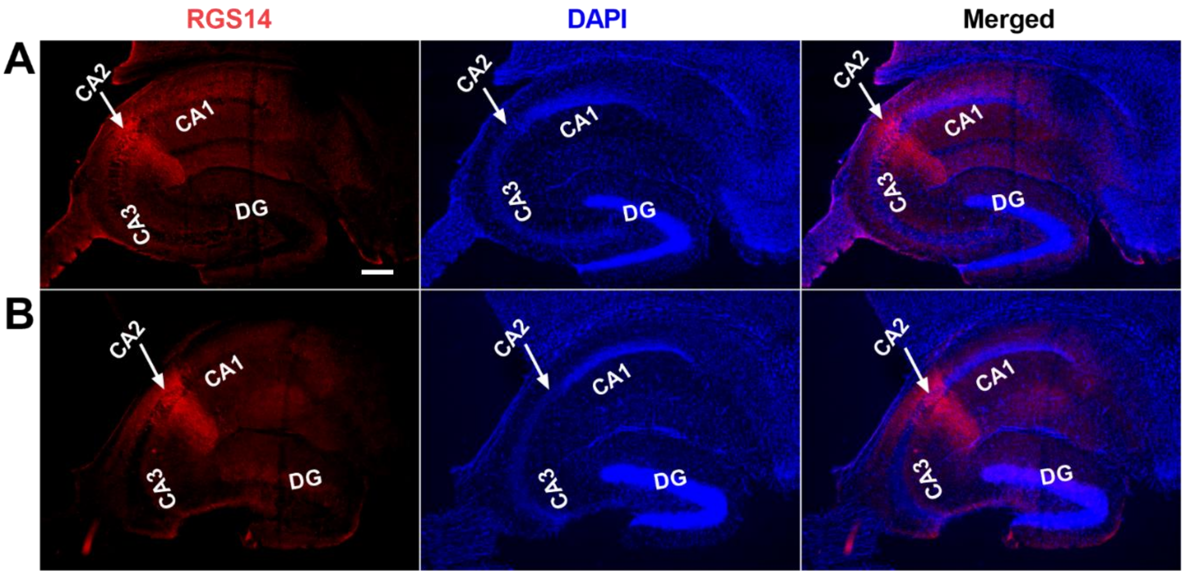

Supplemental Figure S1. Immunostaining of RGS14 as a CA2 marker.

(A-B) Immunofluorescence of transversal hippocampal slices depicting RGS14 expression (red) concentrated in the CA2 region. Nuclei are labeled with DAPI (blue). The horizontal white line indicates a distance of 200 µm. RGS14 immunostaining enables delineation of the CA2 boundaries. In A) a hippocampal slice from *Uhrf*$^{+/+}$ and in B) from *Uhrf*$^{-/-}$ is depicted.

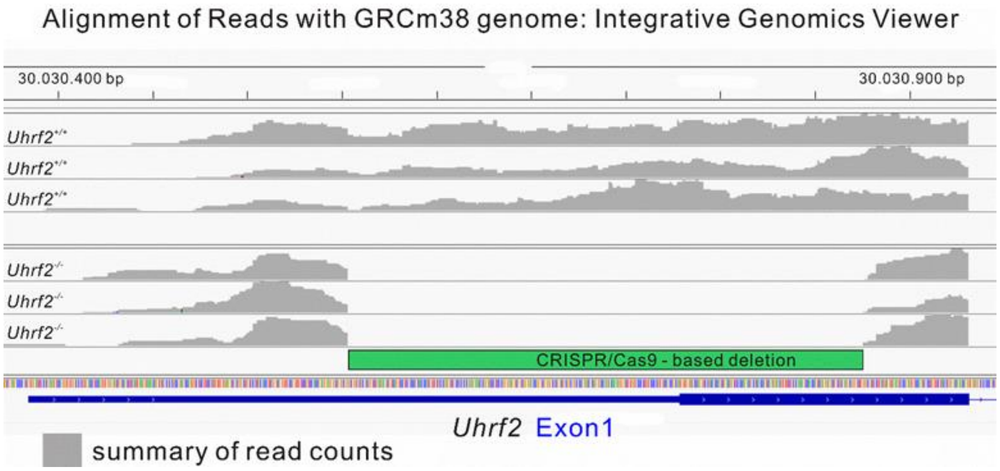

Supplemental Figure S2. RNA sequencing confirms *Uhrf2* exon 1 modification.

Analysis of RNA sequencing reads validates the anticipated deletion (green region) of *Uhrf2* exon 1 in *Uhrf2*$^{-/-}$ mice (n=3) relative to *Uhrf2*$^{+/+}$ wildtype littermate controls (n=3). The deletion of exon 1 sequences is evident in the *Uhrf2* knockouts.
